# Viral replication is enhanced by an HIV-1 intersubtype recombination-derived Vpu protein

**DOI:** 10.1186/1743-422X-7-259

**Published:** 2010-10-04

**Authors:** Cristian De Candia, Constanza Espada, Gabriel Duette, Yanina Ghiglione, Gabriela Turk, Horacio Salomón, Mauricio Carobene

**Affiliations:** 1 National Reference Center for AIDS, Department of Microbiology, School of Medicine, University of Buenos Aires, Buenos Aires, Argentina

## Abstract

**Background:**

Multiple HIV-1 intersubtype recombinants have been identified in human populations. Previous studies from our lab group have shown that the epidemic in Argentina is characterized by the high prevalence of a circulating recombinant form, CRF12_BF, and many related BF recombinant forms. In these genomic structures a recombination breakpoint frequently involved the *vpu *coding region. Due to the scarce knowledge of Vpu participation in the virion release process and its impact on pathogenesis and of the functional capacities of intersubtype recombinant Vpu proteins, the aim of this work was to perform a comparative analysis on virion release capacity and relative replication capacity among viral variants harboring either a BF recombinant Vpu or a subtype B Vpu.

**Results:**

Our results showed that BF recombinant Vpu was associated to an increased viral particles production when compared to WT B variant in tetherin-expressing cell lines. This observation was tested in the context of a competition assay between the above mentioned variants. The results showed that the replication of the BF Vpu-harboring variant was more efficient in cell cultures than subtype B, reaching a higher frequency in the viral population in a short period of time.

**Conclusion:**

This study showed that as a result of intersubtype recombination, a structurally re-organized HIV-1 Vpu has an improved *in vitro *capacity of enhancing viral replication, and provides evidence of the changes occurring in this protein function that could play an important role in the successful spread of intersubtype recombinant variants.

## Background

Multiple HIV-1 intersubtype recombinants have been identified in human populations. To date, more than 40 circulating recombinant forms (CRFs) have been described (Los Alamos HIV Database). In South American countries such as Argentina and Brazil, the epidemic shows considerable diversity in terms of subtypes and CRFs. Previous studies from our lab group have shown that Argentina is characterized by the co-circulation of intersubtype recombinants, mainly BF [1-7] and subtype B viruses. Nucleotide sequence analyses showed that HIV-1 BF recombinants exhibit diverse mosaic structures and that the prototypic CRF12_BF genome mainly corresponds to subtype F1 with five segments of subtype B distributed all along the genome [1,5,6]. In these genomic structures a recombination breakpoint frequently involved the *vpu *coding region. The resulting recombinant protein structure exhibits a subtype B membrane-spanning domain and α- helix I, and a subtype F1 α- helix II.

Regarding protein functions, two distinct biological activities have been attributed to Vpu: a) proteasomal degradation of CD4, and b) enhancement of virion release from infected cells. Although the mechanism by which Vpu degrades CD4 is well delineated [8-10], its enhancing effect on virion release is not fully clarified. It was shown that the transmembrane domain of the protein forms cation-selective ion channels in cellular membranes and that this feature might be involved in facilitating virion release [11,12]. Nevertheless, recent studies have shown that Vpu-dependent virion release is related to its capacity to counteract an INFα-inducible host restriction factor known as Bst-2/CD317/tetherin [13-15].

Since data on Vpu participation in the virion release process and its impact on pathogenesis is still incomplete and scarce, and on account of the remarkable protein diversity, and that most studies have been conducted on Vpu protein from subtypes B or C, the present study was aimed at analyzing the virion release capacity of a naturally occurring BF recombinant Vpu, and its comparison with its pure subtype B counterpart. A chimeric variant harboring a naturally occurring BF recombinant *vpu *coding sequence on a subtype B genetic background (NL4-3) was generated and used for the studies. Our results show that the BF recombinant Vpu is capable of enhancing virion release more efficiently, suggesting an improvement in this function as a consequence of an intersubtype recombination event.

## Results

### Structural characterization of naturally occurring BF intersubtype recombinant Vpu sequences

A previous study from our lab group found a high percentage of BF recombinant strains (56.9%, n = 283) among drug-naïve newly diagnosed HIV-1 individuals [7]. When re-analyzing this group of samples we found that 109 out of 161 (67.7%) of these recombinant variants exhibited a recombination pattern which involved the *vpu *coding region. The analysis of these sequences was performed by means of a tool to detect HIV-1 genomic recombinants available online at http://jphmm.gobics.de/, and confirmed by bootscaning analysis (Simplot V3.5.1). Seventy four (68.3%) sequences presented a very similar recombination pattern to that observed in CRF12_BF, where the breakpoint was located between positions 6195 and 6205 of the genomic sequence, according to the HXB2 numbering.

**Figure 1:**
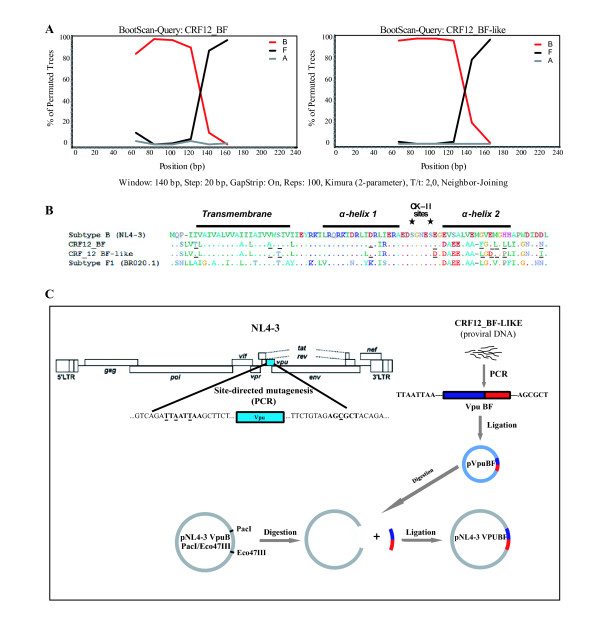
**A. Comparison of bootscaning analysis performed on CRF12_BF and CRF12_BF-like showed a similar recombination pattern, with a breakpoint located between α-helix I and II**. Reference sequences of subtype B (red), F (black), and A (blue) - out group - were obtained from Los Alamos Database. Plots represent bootstrap values based on 100 re-samplings, supporting branching with reference sequences within a 140 nt window moving in steps of 20 bases. The structural domains as well as the phosphorylation sites (asterisks) of Vpu are shown in Figure 1B. Underlined residues represent differences between CRF12_BF and CRF-like variant. C. Diagram depicts the construction step of a chimeric viral genome: *vpu *coding sequence from a naturally occurring BF intersubtype recombinant variant (named CRF12_BF-like) amplified by PCR from a genomic DNA sample obtained from an infected patient. The use of modified oligonucleotides allowed the introduction of both PacI and Eco47III restriction sites. The purified PCR product was cloned into a commercial vector (pGEM-T) to obtain the pVpuBF plasmid. In parallel, these restriction sites were introduced in pNL4-3 by PCR-based site-directed mutagenesis. After digestion and ligation steps a chimeric variant harboring the Vpu BF sequence into the subtype B genomic background (pNL4-3 VpuBF) was obtained.

A naturally occurring BF recombinant was selected for the biological analysis. This variant, named CRF12_BF-like, was chosen because it shares a similar recombination pattern to that observed in the prototypic CRF12_BF [1] (Figure 1A) where the membrane-spanning domain and the α-helix I intracytoplasmic domain (positions 1-50), and the α-helix II together with the 10 C-terminal aminoacids (positions 51-81) correspond to subtypes B and F1 respectively. Comparison of its primary amino acid sequences (CRF12_BF vs. CRF12_BF-like) showed that only 9 out of 81 residues (11.1%) differ from each other, i.e. T6I, A22V, S24T, I43L, E58D, F67L, E69D, L72P, and N80I (Figure 1B). An additional analysis performed on 74 recombinant sequences showed that these substitutions did not occur as inherent features of the selected CRF12_BF-like variant. Even more, some of them were highly represented among the naturally occurring recombinants.

A serine residue, either at position 61 or 64 (S61 or S64) has been shown to be involved in the regulation of Vpu degradation process [16]. Neither S61 nor S64 were present in the CRF12_BF-like variant. Additionally, the analysis of B (n = 137), F1 (n = 12) and BF recombinant (n = 120) Vpu sequences (Los Alamos Database) from Argentina, Brazil, and Uruguay showed that S64 was present in 82% and 6.6% of B and BF sequences respectively. This residue was not found among F1 sequences. None of the whole set of sequences harbored the S61 residue.

The comparative analysis of Vpu amino acid sequences performed among the variants used in this work, in terms of protein domains, is shown in Table 1. Most of the differences were found in the α-helix II and C-terminal domains. Of note, net charge of these domains differed among variants. BF has + 4 and - 5 charges in the α-helix I and α-helix II respectively, compared to the + 3 and - 4 charges found in the subtype B variant. The net charge of C-terminal domains remained constant (-1) although amino acid changes were found.

**Table 1:** Comparative analysis of amino acid differences between subtype B and CRF12_BF-like variants. Positions are shown according to protein domains.

N-Terminal							Transmembrane					α-Helix I					
**Positions**	**3**	**4**	**5**				**7**	**16**	**18**	**24**	**27**	**43**	**46**	**47**			

Subtype B	P	-	I				V	I	I	S	I	I	L	I			

CRF12_BF-like	S	L	V				L	A	L	T	L	L	I	R			

																	
**α-Helix II**												**C-Terminal**					

**Positions**	**58**	**60**	**61**	**62**	**63**	**65**	**66**	**67**	**68**	**69**	**70**	**73**	**74**	**75**	**77**	**78**	**81**

Subtype B	E	E	V	S	A	V	E	M	G	V	E	H	H	A	W	D	D

CRF12_BF-like	D	D	A	E	E	A	A	-	L	G	G	P	L	I	G	N	I

### Functional analysis of BF intersubtype recombinant Vpu

#### *vpu *mRNA is correctly synthesized from the BF Vpu harboring-chimeric variant

A chimeric viral genome, named pNL4-3 VpuBF, harboring the CRF12_BF-like Vpu sequence on a pure subtype B (NL4-3) background (Figure 1C), was generated to evaluate the consequences of intersubtype recombination involving the *vpu *coding region on viral particles release enhancement.

**Figure 2:**
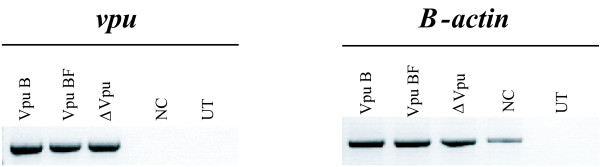
**Vpu expression was assessed by specific RT-PCR of *vpu *mRNA after transfection of HeLa cells**. Both untransfected (UT) cells and cells transfected with empty vector (NC) were used as control (right panel). Amplification of β-*actin *mRNA was also evaluated as an internal control (left panel).

In order to test the *vpu *mRNA expression, HeLa cell cultures were transfected either with pNL4-3 VpuBF, pNL4-3 VpuB and pNL4-3 ΔVpu plasmids. Forty eight hours post-transfection total mRNA content was obtained from each transfected culture, and *vpu *mRNA production was evaluated by qualitative RT-PCR. β-*actin *mRNA was used as control. All transfected cell cultures exhibited comparable expression levels of *vpu *mRNA (Figure 2).

### BF Vpu correlates with an increased viral release in Vpu-dependent cell line models

This study was aimed at evaluating the impact of BF recombinant Vpu on viral release by transfecting both HeLa and 293T cell lines with pNL4-3 VpuBF, pNL4-3 VpuB and pNL4-3 ΔVpu. As previously described, HIV-1 Vpu is essential for viral replication in certain cell lines (e.g. HeLa) but dispensable in others (e.g. 293T) [13-15,17]. Viral production was monitored by quantifying p24 antigen in cell culture supernatants 24 and 48 h post-transfection.

**Figure 3:**
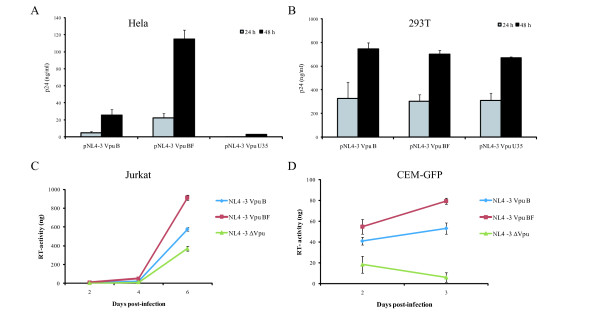
**HeLa (A) and 293T cells (B) were transfected with plasmids pNL4-3 VpuB, pNL4-3 Vpu BF, and pNL4-3 ΔVpu, separately**. Viral production was evaluated, using an ELISA assay, by measuring the Gag p24 antigen content in cell culture supernatants 24 and 48 hours post-transfection. Jurkat cells (**C**) and CEM-GFP (**D**) were infected with equal MOI of NL4-3 Vpu B, NL4-3 Vpu BF and NL4-3 ΔVpu virus stock, and viral replication was monitored by measuring the virus-associated reverse transcriptase activity in culture supernatants. Error bars indicate standard errors. Results are representative of two independent experiments.

In HeLa cells, transfection with pNL4-3 VpuBF resulted in a substantially higher p24 antigen concentration in culture supernatant when compared with that of the subtype B counterpart and ΔVpu, at 24 h post-transfection (22.5 pg/ml, 4.9 pg/ml and 0.4 pg/ml respectively). This difference persisted after 48 h post-transfection (114.7 pg/ml vs. 25.6 pg/ml), showing an approximately 4.5-fold increase in p24 production when the intersubtype recombinant protein was present (Figure 3A).

The evaluation of post-transfection viral production in 293T cell cultures with the variants studied showed no significant differences in the p24 content of culture supernatants (Figure 3B), confirming that both pNL4-3 and pNLVpuBF promoted viral release with similar efficiency in this Vpu-independent cell model.

Viral release was also evaluated in Jurkat and CEM-GFP cell lines, both known to constitutively express tetherin [13,18] and representing Vpu-dependent models. As described in the Materials and Methods section, Jurkat and CEM-GFP cell cultures were infected with each viral stock (NL4-3 VpuBF, NL4-3 VpuB, NL4-3 ΔVpu), and viral production kinetics was evaluated by quantitation of virus-associated RT activity in culture supernatants. After 24 h, comparable levels of infection were achieved for each virus in both Jurkat and CEM-GFP cell cultures, evaluated as percentage of p24 (+) or GFP-expressing cells (FACS) respectively (data not shown).

Viral production was significantly higher for the NL4-3 VpuBF in both Jurkat and CEM-GFP cell cultures (1.5-fold and 1.8-fold respectively), when compared to the wild type variant (Figures 3C and 3D).

### Relative *in vitro *replication capacity

**Figure 4:**
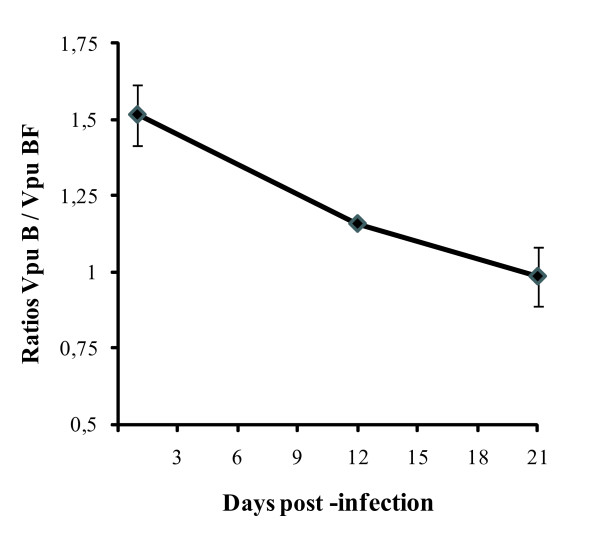
**NL4-3 VpuB and NL4-3 VpuBF viruses were competed against each other by infecting CEM-GFP reporter cells with p24-normalized inocula at 1:1 ratio**. Each variant ratio was determined by clonal analysis of viral population present in culture supernatants. Error bars indicate standard errors. Results are representative of two independent experiments.

The relative replication capacity of WT subtypes B and chimeric BF variants was tested *in vitro *by performing a competitive dual infection. Both variants were used to infect CEM-GFP cell culture, in duplicate, and maintained up to 21 days post-infection (p.i.). Viral population analysis was performed on days 1, 12 and 21 p.i. by cloning and sequencing the *vpu *coding region, as described in the Material and Methods section. Of note, subtype B sequences were predominant at the beginning (B/BF ratio: 1.52) but its ratio changed over time; the recombinant variant became more frequent (B/BF ratio: 0.99) at the end of the experiment. B/BF variants ratio at each time point is depicted in Figure 4.

## Discussion

The *vpu *coding sequence is one of the most variable regions of the HIV-1 genome [19]. It is unknown if this variability and the diversity among different HIV-1 subtypes [20] may have an effect on protein functions, and to what extent this effect may have an impact on HIV-1 transmission and pathogenecity.

A recent report has shown that only HIV-1 strains from the M group express a fully functional Vpu protein and the authors postulate that this may explain the global HIV/AIDS pandemic for the HIV-1 M group [21]. However, most of the structural and functional studies on Vpu have been performed on subtype B proteins. Hence, little is known about the functionality of non-subtype B Vpu, not even about its function as a viral release enhancer. In this work we performed a comparative analysis between subtype B and BF intersubtype recombinant Vpu proteins, focusing on this important function.

The structural analysis carried out on naturally occurring BF recombinant Vpu sequences revealed high conservation of the recombination pattern. This pattern was similar to that observed in the prototypic BF recombinant, CRF12_BF, where the breakpoint is located in the region coding for an amino acid stretch predicted to form a random-coil structure [20]. This region contains two serine residues (S52 and S56), targeted by the casein kinase II, which phosphorylation is an essential step in recruiting the β-TrCP complex and subsequent proteasome-mediated degradation of CD4 [10,22,23]. Recent studies suggest that Vpu may induce internalization and degradation of tetherin by the same pathway [18,24,25].

On the other hand, Vpu degradation is accomplished through a β-TrCP independent mechanism that involves phosphorylation of a serine residue at position 61 or 64. Lack of these residues has been shown to increase the protein stability without affecting the CD4 degradation [16,26]. We have found that S61 was absent in the CRF12_BF and all related BF recombinant Vpu sequences, and that only 6.6% of them presented the S64 residue. Neither CRF12_BF nor the CRF12_BF-like variant harbored S61 or S64, which might suggest that the turnover of the recombinant variants could be different from the pure subtype counterpart, partly explaining at least the higher viral release associated to the former.

It has been shown that an intact transmembrane domain of HIV-1 Vpu is important and sufficient to enhance HIV-1 particle release [12,27]. This domain is a critical determinant of its function since it physically interacts with the BST-2 transmembrane domain leading to its degradation [28,29]. As determined by the comparative analysis of Vpu amino acid sequences performed in this work, 5 amino acid changes were found between the variants studied, and all of the substitutions were conservative, i.e. amino acids belonging to the same chemical group, suggesting a consequent functional conservation of this domain.

Our results showed that BF Vpu increased viral particles production when compared to the WT B variant in tetherin-expressing cell models. As shown in the results obtained from the *in vitro *competition assay, this difference in viral production might represent an advantage for the virus in terms of replication capacity or fitness.

According to the structural characteristics of the recombinant protein, the interchange of the second half of the Vpu coding sequence, encompassing the α-helix II and the C-terminal segment between subtypes B and F1, may have resulted in an alternative protein form with enhanced regulation capacity of the host cell environment.

Previous molecular studies from our lab group have shown that BF intersubtype recombination frequently affected genomic regions involved in regulating viral gene expression, replication, and interaction with the host immune system [30-35]. Taken together, these results sustain the idea that this phenomenon might have played an important role in the successful spread of the resulting variants, arguing in favor of the intersubtype recombination as a powerful source of variability and adaptation capacity.

As accessory proteins and/or their cellular counterparts are nowadays seen as promising future therapeutic targets [36-39], results presented here contribute to the comprehensive and profound knowledge of protein functions and its variations needed to develop more effective and specific therapeutic agents aimed at blocking viral replication.

## Conclusion

As described above, our results show that intersubtype recombination gave rise to a structurally re-organized HIV-1 Vpu variant with an improved capacity of enhancing viral replication, when tested *in vitro*. Although these results correspond to a single recombinant isolate, it provides evidence on the changes in the protein function that could play a role in the spread of intersubtype recombinant variants, having implications on HIV-1 epidemiology and pathogenesis. These data are also important in the context of future therapeutic approaches.

## Methods

### Plasmids, cells and viruses

The *vpu *coding sequence from a naturally occurring BF intersubtype recombinant variant (named CRF12_BF-like) was amplified by PCR from a genomic DNA sample obtained from an infected patient identified in a previous study conducted in Argentina [7]. PCR reaction was carried out by using a high fidelity DNA polymerase (Platinum^® ^Taq DNA Polymerase High Fidelity, Invitrogen). Oligonucleotides used for the reaction were:

VpuBF/PacI (5'-**T**T**A**AT**T**AAGCTTCTCTATCAAAGC-3')

VpuBF/Eco47III (5'-AG**C**GCTAAAGAAAAATTGTGGGTC-3')

Modifications in the oligonucleotides allowed obtaining a PCR product harboring both PacI and Eco47III restriction sites at the 5' and 3' ends of the *vpu *coding sequence respectively. The amplicon was subcloned into a commercial vector (pGEM-T^® ^easy vector, Promega) according to the manufacturer's instructions. The resulting transformants were screened for the insert and the plasmids were sequenced to ensure that BF recombinant *vpu *sequences and restriction sites were intact. The resulting plasmid was named as pVpuBF.

Insertion of the BF *vpu *gene into HIV-1 molecular clone pNL4-3 [40] (obtained from Dr Malcolm Martin through the AIDS Research and Reference Reagent Program, Division of AIDS, NIAID, NIH) was accomplished in a two-step procedure. The first step involved the introduction of PacI and Eco47III restriction site at the 5' and 3' ends of the pNL4-3 *vpu *coding sequence respectively, by site-directed mutagenesis. Oligonucleotides used for this step were:

VpuB/PacI (5'-GCTCATCAGAACAGTCAGA**T**T**A**AT**T**AAGCTTCTCTATCAAAGC-3')

VpuB/Eco47III (5'-GGGATATTGATGATCTGTAG**C**GCTACAGAAAAATTGTGGGTC-3')

The second step consisted in the replacement of the WT pNL4-3 Vpu coding sequence with the CRF12_BF-like Vpu sequence, followed by reversion of the changes introduced to the original sequence by a new cycle of site-directed mutagenesis. Briefly, both pNL4-3 PacI/Eco47III and pVpuBF were digested with each restriction enzyme. Ligation of the pNL4-3 PacI/Eco47III backbone and the VpuBF PacI/Eco47III insert was performed by T4 ligase (Invitrogen). Oligonucleotides used for the site-directed mutagenesis reaction were:

VpuPacIrevt (5'-GCTCATCAGAACAGTCAGA**C**T**C**AT**C**AAGCTTCTCTATCAAAGC-3')

VpuEco47IIIrevt (5'-GGGATATTGATGATCTGTAG**T**GCTACAGAAAAATTGTGGGTC-3')

Again, the resulting transformants were screened for the insert and any possible unwanted changes in the generated plasmid.

Mini and midipreps of selected clones were obtained (QIAgen).

All mutagenesis reactions were performed by using a highly processive DNA polymerase with proofreading 3'-5' exonuclease activity (Platinum^®^PfxDNA Polymerase, Invitrogen). Sequencing was performed on an automatic sequencer (Applied Biosystems DNA sequencer 3100) by using the Big Dye Terminator sequencing kit (Amersham, Sweden). Nucleotide sequences were analyzed and manually adjusted using Sequencher 4.0.5 software (Gene Codes Co, USA).

The Vpu-deficient plasmid pNL4-3 ΔVpu[41] (obtained from Dr. Klaus Strebel through the AIDS Research and Reference Reagent Program, Division of AIDS, NIAID, NIH) contains a defective *vpu *gene, but is otherwise identical to the infectious molecular clone pNL4-3.

HeLa, 293T, MT2, Jurkat T and CEM-GFP cells were used in this study. HeLa and 293T were cultured in DMEM medium (Gibco^®^) supplemented with 10% fetal bovine serum (FBS, Gibco^®^), 2 mM L-glutamine (Gibco^®^), penicillin/streptomycin (100 IU/ml and 100 μg/ml respectively, Gibco^®^). MT2 and Jurkat T cells were cultured in RPMI-1640 medium (Gibco^®^) supplemented with 10% FBS, 2 mM L-glutamine, 100 U/ml penicillin and 100 μg/ml streptomycin. CEM-GFP cells were cultured in the same medium plus 500 μg/ml G418. Cell lines were obtained through the AIDS Research and Reference Reagent Program.

293T cells transient transfections were carried out using Lipofectamine2000 (Invitrogen) following manufacturer's instructions. For single transfections experiments confluent cells grown in 24-well dish were transfected with 0.8 μg of pNL4-3 Vpu B, pNL4-3 VpuBF and pNL4-3 ΔVpu.

Viral stocks were obtained by infecting MT2 cells. Briefly, 2×10^6 ^cells were infected with 1 ml of culture supernatant from 293T cell culture transfections. Afterwards, cells were washed twice with PBS and maintained in culture medium, until CPE was observed. Viral stock titrations (TCID 50/ml values) were performed by using the Reed and Muench method [42].

### RT-PCR

HeLa cells transient transfections were carried out using Lipofectamine2000 (Invitrogen) following manufacturer's instructions. For single transfections experiments confluent cells grown in 60 mm Petri dishes were transfected with 8 μg of pNL4-3 Vpu B, pNL4-3 Vpu BF and pNL4-3 ΔVpu. Untransfected cells as well as cells transfected with empty vectors were always used as negative controls.

Total cellular RNA was extracted from 10^7 ^cells using Trizol^® ^reagent (Invitrogen). Three μg of RNA were reverse transcribed using MMLV reverse transcriptase (Invitrogen) and an oligo-dT primer. The mix was supplemented with RNAse inhibitors (Invitrogen) in a final volume of 20 μl. Two μl of cDNA were used for vpu PCR amplification using the following oligonucleotides:

VpuB fw (5'-CCTCTAGATAATGCAACCTATAATAG-3');

VpuB rev (5'-CACGCGTCTACAGATCATCAATATCC-3');

VpuBF fw (5'-CCTCTAGATAATGCAATCTTTAG-3') and

VpuBF rev (5'-CACGCGTCTACAGAATATCAATATTC-3').

PCR amplification of β-*actin*, used as RNA quality control, was performed by using the following oligonucleotides:

β1 (5'-GGACCTGACTGACTACCTCATGAA-3')

β2 (5'-GATCCACATCTGCTGGAAGGTGG-3').

### Vpu recombinant sequences analysis

BF recombinant Vpu sequences were obtained from Los Alamos HIV sequence database. A multiple alignment with reference sequences was performed using Clustal W, and visually corrected with the BioEdit version 5.0.9 program http://www.mbio.ncsu.edu/BioEdit/bioedit.html.

Detection of intersubtype genomic recombination was carried out by a tool available online at http://jphmm.gobics.de. This method uses a probabilistic approach to compare a sequence to a multiple alignment of a sequence family [43]. Recombination analysis was confirmed by bootscanning analysis as implemented in the SimPlot v.3.5.1 program.

### HIV-1 production assay: transfections and infections

HeLa and 293T cells were transfected with 0.8 μg of pNL4-3 VpuB, pNL4-3 VpuBF and pNL4-3 ΔVpu together with 0.05 μg of GFP expression vector used as a control for the transfection, using Lipoafectamine2000 (Invitrogen) and following manufacture's instruction. Cell culture supernatants were collected 24 and 48 hours post-transfection. HIV-1 p24 antigen quantitation was performed by means of a commercial ELISA assay (Murex, Abbott).

For viral replication kinetics analysis, 10^6 ^logarithmically growing Jurkat or CEM-GFP suspension cells were infected in duplicate with each viral stock (NL4-3 VpuB, NL4-3 VpuBF and NL4-3 ΔVpu) at MOI of 0.01. Afterwards, cells were washed twice with PBS and maintained in culture medium. Cell culture supernatants were harvested at different time points and assayed for RT activity, using the Reverse Transcriptase Colorimetric Assay (Roche) following manufacture's instruction.

### Growth competition assay

Competitive growth of NL4-3 Vpu B and NL4-3 Vpu BF viruses was analyzed as recently described [44]. Briefly, 2×10^6 ^CEM cells were infected in duplicate with wild-type and the BF chimeric variants at a ratio 1:1. Cells samples were collected at 3 time points (1, 12 and 21 d.p.i.), and genomic DNA was extracted using the QIAamp DNA Mini kit (QIAgen). *Vpu *coding sequence was PCR amplified from proviral DNA with primers ACC7 (5'-CTATGGCAGGAAGAAGCGGAGA-3') and ZM140E (5'-GGGGTCAACTTTACACATGGCTTT-3'). PCR products were gel-purified (QIAquick gel extraction kit) and cloned into a commercial cloning vector (pGEMT-EASY^®^, Promega). Nucleotide sequencing was performed as described above.

### Statistical analysis

All data was expressed as mean ± SD unless otherwise stated. Significance (*p *< 0.05) between means of experiments was evaluated using the Student's t test for independence samples (Primer of Biostatistics version 4.02 software)

## Competing interests

The authors declare that they have no competing interests.

## Authors' contributions

CDC contributed to the design of the study, to perform the experiments, and to write the manuscript. GD and YG contributed to perform the experiments. CE provided technical support during cloning and sequencing procedures. GT and HS contributed to design the study, data interpretation and discussion. MC contributed to design the study, to supervise experimental design, data interpretation and discussion and to write the manuscript. All authors read and approved the final manuscript.

## References

[B1] Carr JK Avila M Gomez Carrillo M Salomon H Hierholzer J Watanaveeradej V Pando MA Negrete M Russell KL Sanchez J Diverse BF recombinants have spread widely since the introduction of HIV-1 into South America Aids 2001 15 F41 47 10.1097/00002030-200110190-00002 11600844

[B2] Avila MM Pando MA Carrion G Peralta LM Salomon H Carrillo MG Sanchez J Maulen S Hierholzer J Marinello M Two HIV-1 epidemics in Argentina: different genetic subtypes associated with different risk groups J Acquir Immune Defic Syndr 2002 29 422 426 11917249 10.1097/00126334-200204010-00015

[B3] Gomez Carrillo M Avila M Hierholzer J Pando M Martinez PL McCutchan FE Carr JK Mother-to-child HIV type 1 transmission in Argentina: BF recombinants have predominated in infected children since the mid-1980s AIDS Res Hum Retroviruses 2002 18 477 483 10.1089/088922202317406619 12015900

[B4] Thomson MM Delgado E Herrero I Villahermosa ML Vazquez-de Parga E Cuevas MT Carmona R Medrano L Perez-Alvarez L Cuevas L Najera R Diversity of mosaic structures and common ancestry of human immunodeficiency virus type 1 BF intersubtype recombinant viruses from Argentina revealed by analysis of near full-length genome sequences J Gen Virol 2002 83 107 119 11752707 10.1099/0022-1317-83-1-107

[B5] Quarleri JF Rubio A Carobene M Turk G Vignoles M Harrigan RP Montaner JS Salomon H Gomez-Carrillo M HIV type 1 BF recombinant strains exhibit different pol gene mosaic patterns: descriptive analysis from 284 patients under treatment failure AIDS Res Hum Retroviruses 2004 20 1100 1107 10.1089/aid.2004.20.1100 15585101

[B6] Thomson MM Sierra M Tanuri A May S Casado G Manjon N Najera R Analysis of near full-length genome sequences of HIV type 1 BF intersubtype recombinant viruses from Brazil reveals their independent origins and their lack of relationship to CRF12_BF AIDS Res Hum Retroviruses 2004 20 1126 1133 10.1089/aid.2004.20.1126 15585105

[B7] Dilernia DA Gomez AM Lourtau L Marone R Losso MH Salomon H Gomez-Carrillo M HIV type 1 genetic diversity surveillance among newly diagnosed individuals from 2003 to 2005 in Buenos Aires, Argentina AIDS Res Hum Retroviruses 2007 23 1201 1207 10.1089/aid.2007.0068 17961105

[B8] Willey RL Maldarelli F Martin MA Strebel K Human immunodeficiency virus type 1 Vpu protein induces rapid degradation of CD4 J Virol 1992 66 7193 7200 1433512 10.1128/jvi.66.12.7193-7200.1992 PMC240416

[B9] Willey RL Maldarelli F Martin MA Strebel K Human immunodeficiency virus type 1 Vpu protein regulates the formation of intracellular gp160-CD4 complexes J Virol 1992 66 226 234 1727486 10.1128/jvi.66.1.226-234.1992 PMC238279

[B10] Binette J Dube M Mercier J Halawani D Latterich M Cohen EA Requirements for the selective degradation of CD4 receptor molecules by the human immunodeficiency virus type 1 Vpu protein in the endoplasmic reticulum Retrovirology 2007 4 75 10.1186/1742-4690-4-75 17937819 PMC2170451

[B11] Ewart GD Sutherland T Gage PW Cox GB The Vpu protein of human immunodeficiency virus type 1 forms cation-selective ion channels J Virol 1996 70 7108 7115 8794357 10.1128/jvi.70.10.7108-7115.1996 PMC190763

[B12] Schubert U Ferrer-Montiel AV Oblatt-Montal M Henklein P Strebel K Montal M Identification of an ion channel activity of the Vpu transmembrane domain and its involvement in the regulation of virus release from HIV-1-infected cells FEBS Lett 1996 398 12 18 10.1016/S0014-5793(96)01146-5 8946945

[B13] Neil SJ Sandrin V Sundquist WI Bieniasz PD An interferon-alpha-induced tethering mechanism inhibits HIV-1 and Ebola virus particle release but is counteracted by the HIV-1 Vpu protein Cell Host Microbe 2007 2 193 203 10.1016/j.chom.2007.08.001 18005734 PMC3793644

[B14] Neil SJ Zang T Bieniasz PD Tetherin inhibits retrovirus release and is antagonized by HIV-1 Vpu Nature 2008 451 425 430 10.1038/nature06553 18200009

[B15] Van Damme N Goff D Katsura C Jorgenson RL Mitchell R Johnson MC Stephens EB Guatelli J The interferon-induced protein BST-2 restricts HIV-1 release and is downregulated from the cell surface by the viral Vpu protein Cell Host Microbe 2008 3 245 252 10.1016/j.chom.2008.03.001 18342597 PMC2474773

[B16] Estrabaud E Le Rouzic E Lopez-Verges S Morel M Belaidouni N Benarous R Transy C Berlioz-Torrent C Margottin-Goguet F Regulated degradation of the HIV-1 Vpu protein through a betaTrCP-independent pathway limits the release of viral particles PLoS Pathog 2007 3 e104 10.1371/journal.ppat.0030104 17676996 PMC1933454

[B17] Varthakavi V Smith RM Bour SP Strebel K Spearman P Viral protein U counteracts a human host cell restriction that inhibits HIV-1 particle production Proc Natl Acad Sci USA 2003 100 15154 15159 10.1073/pnas.2433165100 14657387 PMC299932

[B18] Douglas JL Viswanathan K McCarroll MN Gustin JK Fruh K Moses AV Vpu directs the degradation of the human immunodeficiency virus restriction factor BST-2/Tetherin via a {beta}TrCP-dependent mechanism J Virol 2009 83 7931 7947 10.1128/JVI.00242-09 19515779 PMC2715753

[B19] Yusim K Kesmir C Gaschen B Addo MM Altfeld M Brunak S Chigaev A Detours V Korber BT Clustering patterns of cytotoxic T-lymphocyte epitopes in human immunodeficiency virus type 1 (HIV-1) proteins reveal imprints of immune evasion on HIV-1 global variation J Virol 2002 76 8757 8768 10.1128/JVI.76.17.8757-8768.2002 12163596 PMC136996

[B20] McCormick-Davis C Dalton SB Singh DK Stephens EB Comparison of Vpu sequences from diverse geographical isolates of HIV type 1 identifies the presence of highly variable domains, additional invariant amino acids, and a signature sequence motif common to subtype C isolates AIDS Res Hum Retroviruses 2000 16 1089 1095 10.1089/08892220050075363 10933625

[B21] Sauter D Schindler M Specht A Landford WN Munch J Kim KA Votteler J Schubert U Bibollet-Ruche F Keele BF Tetherin-driven adaptation of Vpu and Nef function and the evolution of pandemic and nonpandemic HIV-1 strains Cell Host Microbe 2009 6 409 421 10.1016/j.chom.2009.10.004 19917496 PMC2779047

[B22] Margottin F Bour SP Durand H Selig L Benichou S Richard V Thomas D Strebel K Benarous R A novel human WD protein, h-beta TrCp, that interacts with HIV-1 Vpu connects CD4 to the ER degradation pathway through an F-box motif Mol Cell 1998 1 565 574 10.1016/S1097-2765(00)80056-8 9660940

[B23] Meusser B Sommer T Vpu-mediated degradation of CD4 reconstituted in yeast reveals mechanistic differences to cellular ER-associated protein degradation Mol Cell 2004 14 247 258 10.1016/S1097-2765(04)00212-6 15099523

[B24] Mangeat B Gers-Huber G Lehmann M Zufferey M Luban J Piguet V HIV-1 Vpu neutralizes the antiviral factor Tetherin/BST-2 by binding it and directing its beta-TrCP2-dependent degradation PLoS Pathog 2009 5 e1000574 10.1371/journal.ppat.1000574 19730691 PMC2729927

[B25] Mitchell RS Katsura C Skasko MA Fitzpatrick K Lau D Ruiz A Stephens EB Margottin-Goguet F Benarous R Guatelli JC Vpu antagonizes BST-2-mediated restriction of HIV-1 release via beta-TrCP and endo-lysosomal trafficking PLoS Pathog 2009 5 e1000450 10.1371/journal.ppat.1000450 19478868 PMC2679223

[B26] Hill MS Ruiz A Schmitt K Stephens EB Identification of amino acids within the second alpha helical domain of the human immunodeficiency virus type 1 Vpu that are critical for preventing CD4 cell surface expression Virology 397 104 112 10.1016/j.virol.2009.10.048 19944437 PMC4104991

[B27] Paul M Mazumder S Raja N Jabbar MA Mutational analysis of the human immunodeficiency virus type 1 Vpu transmembrane domain that promotes the enhanced release of virus-like particles from the plasma membrane of mammalian cells J Virol 1998 72 1270 1279 9445027 10.1128/jvi.72.2.1270-1279.1998 PMC124605

[B28] Iwabu Y Fujita H Kinomoto M Kaneko K Ishizaka Y Tanaka Y Sata T Tokunaga K HIV-1 accessory protein Vpu internalizes cell-surface BST-2/tetherin through transmembrane interactions leading to lysosomes J Biol Chem 2009 284 35060 35072 10.1074/jbc.M109.058305 19837671 PMC2787367

[B29] McNatt MW Zang T Hatziioannou T Bartlett M Fofana IB Johnson WE Neil SJ Bieniasz PD Species-specific activity of HIV-1 Vpu and positive selection of tetherin transmembrane domain variants PLoS Pathog 2009 5 e1000300 10.1371/journal.ppat.1000300 19214216 PMC2633611

[B30] Carobene MG Rodrigues CR De Candia CA Turk G Salomon H In vitro dynamics of HIV-1 BF intersubtype recombinants genomic regions involved in the regulation of gene expression Virol J 2009 6 107 10.1186/1743-422X-6-107 19607724 PMC2717942

[B31] Carobene MG Rubio AE Carrillo MG Maligne GE Kijak GH Quarleri JF Salomon H Differences in frequencies of drug resistance-associated mutations in the HIV-1 pol gene of B subtype and BF intersubtype recombinant samples J Acquir Immune Defic Syndr 2004 35 207 209 10.1097/00126334-200402010-00018 14722457

[B32] Rodriguez AM Turk G Pascutti MF Ferrer F Najera JL Monaco D Esteban M Salomon H Calamante G Gherardi MM Characterization of DNA and MVA vectors expressing Nef from HIV-1 CRF12_BF revealed high immune specificity with low cross-reactivity against subtype B Virus Res 2009 146 1 12 10.1016/j.virusres.2009.08.004 19715734

[B33] Turk G Carobene M Monczor A Rubio AE Gomez-Carrillo M Salomon H Higher transactivation activity associated with LTR and Tat elements from HIV-1 BF intersubtype recombinant variants Retrovirology 2006 3 14 10.1186/1742-4690-3-14 16483381 PMC1402313

[B34] Turk G Gherardi MM Laufer N Saracco M Luzzi R Cox JH Cahn P Salomon H Magnitude, breadth, and functional profile of T-cell responses during human immunodeficiency virus primary infection with B and BF viral variants J Virol 2008 82 2853 2866 10.1128/JVI.02260-07 18184702 PMC2258999

[B35] Turk G Gundlach S Carobene M Schindler M Salomon H Benaroch P Single Nef proteins from HIV type 1 subtypes C and F fail to upregulate invariant chain cell surface expression but are active for other functions AIDS Res Hum Retroviruses 2009 25 285 296 10.1089/aid.2008.0132 19327048

[B36] Aida Y Matsuda G Role of Vpr in HIV-1 nuclear import: therapeutic implications Curr HIV Res 2009 7 136 143 10.2174/157016209787581418 19275582

[B37] Foster JL Garcia JV HIV-1 Nef: at the crossroads Retrovirology 2008 5 84 10.1186/1742-4690-5-84 18808677 PMC2563024

[B38] Montal M Vpu matchmakers as a therapeutic strategy for HIV infection PLoS Pathog 2009 5 e1000246 10.1371/journal.ppat.1000246 19478874 PMC2680973

[B39] Strebel K Luban J Jeang KT Human cellular restriction factors that target HIV-1 replication BMC Med 2009 7 48 10.1186/1741-7015-7-48 19758442 PMC2759957

[B40] Adachi A Gendelman HE Koenig S Folks T Willey R Rabson A Martin MA Production of acquired immunodeficiency syndrome-associated retrovirus in human and nonhuman cells transfected with an infectious molecular clone J Virol 1986 59 284 291 3016298 10.1128/jvi.59.2.284-291.1986 PMC253077

[B41] Strebel K Klimkait T Martin MA A novel gene of HIV-1, vpu, and its 16-kilodalton product Science 1988 241 1221 1223 10.1126/science.3261888 3261888

[B42] Reed LJMH A simple method of estimating fifty percent endpoints Am J Hyg 1938 27 493 497

[B43] Schultz AK Zhang M Leitner T Kuiken C Korber B Morgenstern B Stanke M A jumping profile Hidden Markov Model and applications to recombination sites in HIV and HCV genomes BMC Bioinformatics 2006 7 265 10.1186/1471-2105-7-265 16716226 PMC1525204

[B44] Ludwig C Leiherer A Wagner R Importance of protease cleavage sites within and flanking human immunodeficiency virus type 1 transframe protein p6* for spatiotemporal regulation of protease activation J Virol 2008 82 4573 4584 10.1128/JVI.02353-07 18321978 PMC2293064

